# Unveiling the Effects of Hydroxyl‐Induced Trap States on the Charge Transport in p‐ and n‐Channel Organic Field‐Effect Transistors through Variable‐Temperature Characterization

**DOI:** 10.1002/adma.202505631

**Published:** 2025-06-12

**Authors:** Yurii Radiev, Tobias Wollandt, Hagen Klauk, Gregor Witte

**Affiliations:** ^1^ Philipps‐Universität Marburg Renthof 7 35032 Marburg Germany; ^2^ Max Planck Institute for Solid State Research Heisenbergstr. 1 70569 Stuttgart Germany

**Keywords:** activation energy, contact resistance, interfacial trap states, organic field‐effect transistor, Schottky barrier, transfer length method, variable‐temperature analysis

## Abstract

Trap states at the gate dielectric‐organic semiconductor (OSC) interface are one of the main sources of extrinsic traps in organic field‐effect transistors (OFETs). However, they are often overlooked and their effects on the charge transport are attributed to the exposure of devices to ambient air. Here a first variable‐temperature transfer length method characterization of both p‐ and n‐channel OFETs under full high vacuum conditions is reported. By comparing a hydroxylated aluminum oxide (Al_2_O_3_) gate dielectric with a hydroxyl‐free, tetradecylphosphonic acid‐functionalized Al_2_O_3_ dielectric, it is shown that hydroxyl‐induced trap states reduce the charge carrier mobility in OFETs regardless of the channel type. This observation challenges the common belief that the hydroxyl‐induced traps are affecting primarily the n‐channel transport. The variable‐temperature analysis yields a high activation energy of charge transport as the main effect of a hydroxylated gate dielectric. Moreover, the injection barrier at the interface between the source‐drain electrodes and the OSC layer is significantly lower for devices with a hydroxyl‐free dielectric and correlates with the activation energy of charge transport. This work identifies previously hidden limitations of charge transport in OFETs, opening opportunities for further improvements in device performance and potential device applications.

## Graphical abstract

A full high vacuum preparation and characterization chain unveils that hydroxyl‐induced trap states affect the charge transport in p‐ and n‐channel OFETs similarly. The variable‐temperature TLM analysis suggests that the activation energy of charge transport plays a more significant role than the density of trap states. Furthermore, the injection barrier is significantly lower in devices with a hydroxyl‐free dielectric.

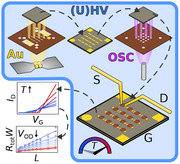

## 1 Introduction

**Scheme 1:**
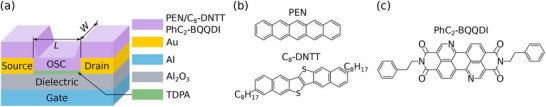
a) Bottom contact‐bottom gate OFET geometry used in this study (see Section 4 for more details). b,c) Representation of the molecular structure of organic semiconductors used for p‐ and n‐channel OFETs, respectively.

Organic semiconductors (OSCs) are a versatile class of materials that are currently not only being investigated in research but are already used in commercial applications such as light‐emitting diodes and displays,^[^ ^1^ ^]^ and have a great potential for applications in photovoltaics,^[^ ^2^, ^3^ ^]^ printable and wearable electronics,^[^ ^4^, ^5^, ^6^ ^]^ as well as bio‐sensors and lab‐on‐chip devices.^[^ ^7^, ^8^, ^9^, ^10^ ^]^ For a long time, the low charge carrier mobility of OSCs – compared to inorganic semiconductors – was considered a major obstacle to the widespread use of organic electronic devices, such as organic field‐effect transistors (OFETs). In thin films of OSCs, which are most relevant for organic electronics,^[^ ^11^, ^12^, ^13^, ^14^ ^]^ extrinsic sources of trap states, such as heterogeneous interfaces, environmental exposure and grain boundaries, have a dominant impact on charge transport.^[^ ^15^, ^16^, ^17^, ^18^, ^19^, ^20^, ^21^, ^22^, ^23^ ^]^ Nowadays, the interface between the gate dielectric and the OSC active layer is considered to be one of the largest sources of extrinsic trap states in OFETs.^[^ ^23^, ^24^, ^25^, ^26^ ^]^ To date, however, most of the research on trap states and contact resistance in OFETs has been performed using p‐channel OSCs,^[^ ^23^, ^26^, ^27^, ^28^, ^29^, ^30^, ^31^ ^]^ in particular because for a long time n‐channel conductivity has been difficult to observe in devices with hydroxylated gate dielectrics.^[^ ^23^, ^32^, ^33^ ^]^ Some insight into this problem was provided by Chua et al., who showed in their work that by using a hydroxyl‐free gate dielectric, n‐channel OFET conduction with many conjugated polymers can be achieved. They therefore concluded that hydroxyl groups on the surface of dielectrics form trap states that lead to strong trapping of electrons, but not of holes.^[^ ^32^ ^]^ This explanation has become widely accepted, soon transforming into the belief that hydroxyl groups are primarily electron‐trapping.^[^ ^23^, ^32^, ^33^ ^]^ Since then, many studies reporting a low charge carrier mobility in p‐channel OFETs attributed the latter to negative effects of ambient exposure on the OSC films, ignoring the hydroxylated surface of the gate dielectric.^[^ ^34^, ^35^ ^]^ However, the extent to which the electronic characteristics of p‐channel OSCs change upon exposure to moisture and/or oxygen is typically much smaller than what is reported for OFETs with hydroxylated gate dielectrics.^[^ ^36^, ^37^, ^38^, ^39^, ^40^ ^]^ Especially with regard to the recent developments in synthesizing high‐charge carrier mobility OSCs, the question arises: Do hydroxyl groups on the surface of common gate dielectrics trap strongly not only electrons, but also holes, thus significantly limiting the performance of p‐channel OFETs? To address this question we developed a full‐high vacuum (HV) processing and characterization chain that excludes ambient exposure of OFETs after the deposition of the active layer. We use a bottom gate‐bottom contact (BGBC) device architecture to ensure well‐defined interfaces with gate dielectric and source‐drain electrodes (see Scheme **1**a). A tetradecylphosphonic acid (TDPA)‐functionalized Al_2_O_3_ dielectric serves as a hydroxyl‐free surface, which allows a direct comparison with a hydroxylated (bare) Al_2_O_3_. Pentacene (PEN) is chosen as an extensively‐studied p‐channel model system and 2,9dioctylnaphtho[2,3‐b]naphtha[2',3':4,5]thieno[2,3‐d]thiophene (C_8_‐DNTT) as its modern high‐performance successor (Scheme 1b). The n‐channel devices are based on phenylalkyl‐substituted 3,4,9,10benzo[*de*]isoquinolino[1,8*gh*]quinolinetetracarboxylic diimide (PhC_2_‐BQQDI) (Scheme 1c), chosen due to its superb charge transport properties and possibility to be deposited via organic molecular‐beam deposition (OMBD).^[^ ^41^, ^42^ ^]^ The full‐HV processing chain allows us to directly compare p‐ and n‐channel OFETs with hydroxylated and hydroxyl‐free gate dielectrics. Moreover, a variable‐temperature transfer length method (TLM) analysis makes it possible to probe otherwise inaccessible charge transport parameters, such as the activation energy of charge carrier transport, the energetic density of trap states at the gate dielectric‐OSC interface and the injection barrier at the electrode‐OSC interface. Combining these techniques with the ability to monitor the work function of the electrodes, we are able to unveil how exactly the hydroxyl‐induced trap states affect the charge transport in both p‐ and n‐channel OFETs.

**Figure 1:**
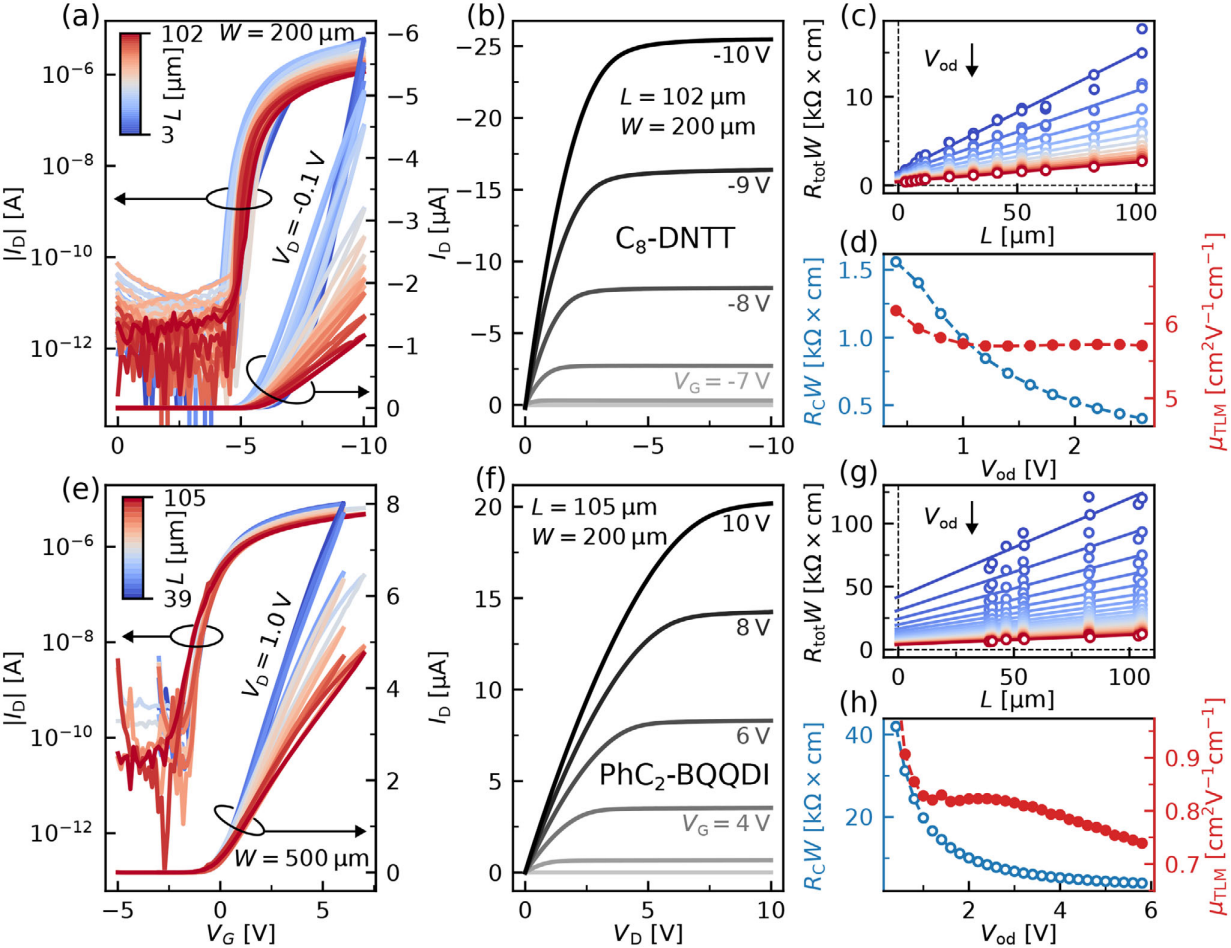
Exemplary OFET characteristics and room temperature TLM analysis of C_8_‐DNTT and PhC_2_‐BQQDI OFETs, respectively: a,e) Transfer characteristics in the linear regime of operation for a set of devices with varying channel lengths *L*. b,f) Output characteristics of a single device. c,g) TLM plot constructed using transfer characteristics from (a) and (e). The overdrive voltage *V*_od_ increases from the top curve to the bottom one (color‐coded blue to red); solid lines are least‐squares fits to the data. d,h) Contact resistance *R*_C_ *W* (empty circles, blue) and charge carrier mobility µ_TLM_ (filled circles, red) extracted from TLM plots in (c) and (g); the dashed lines through the data points are guides to the eye only. All OFETs are prepared with a TDPA‐functionalized Al_2_O_3_ gate dielectric with a thickness of 20 nm.

**Figure 2:**
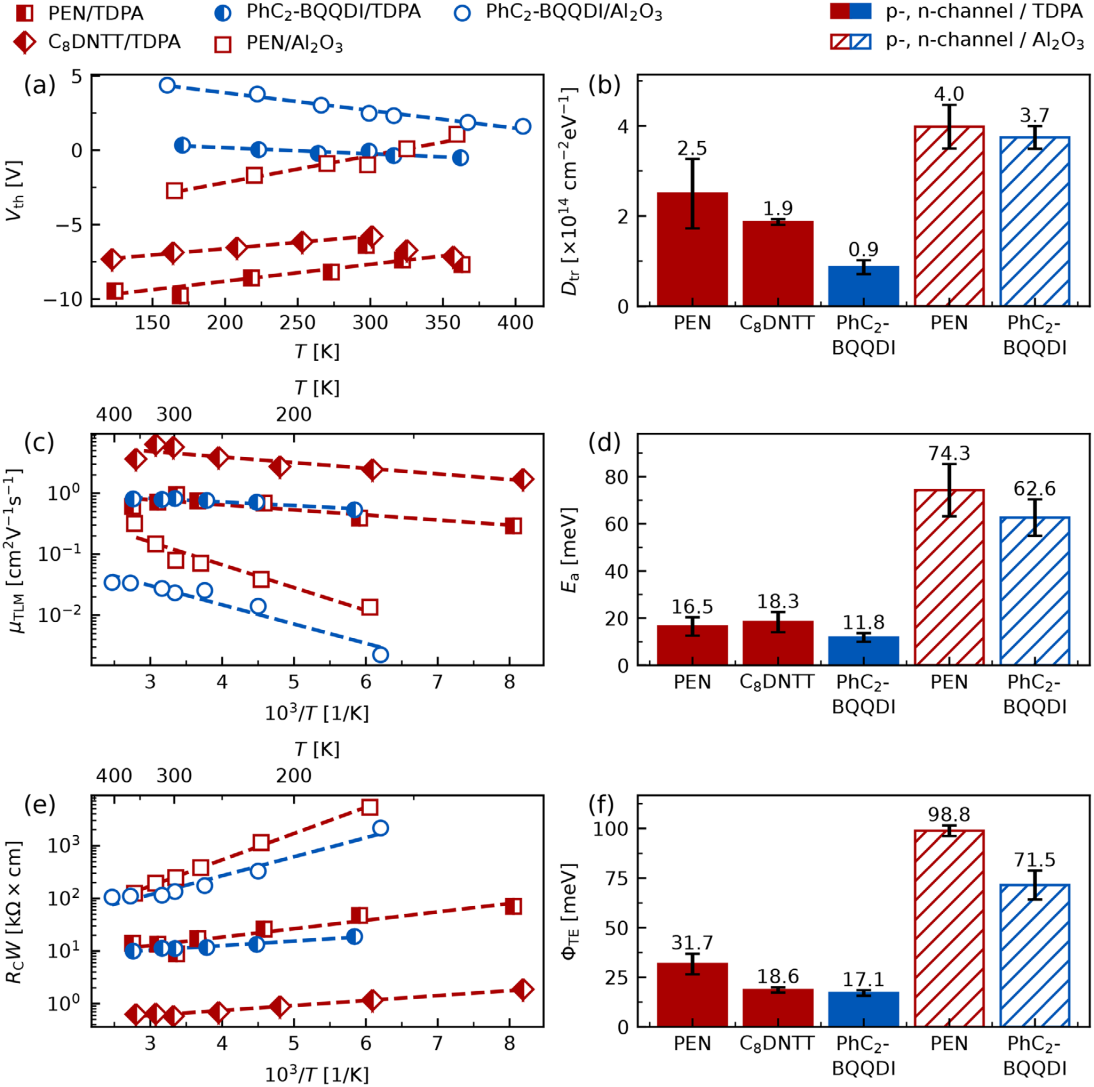
Results of the variable‐temperature analysis of the OFET characteristics: a) Temperature dependence of threshold voltages *V*_th_, (c) Arrhenius plot of TLM charge carrier mobilities µ_TLM_ and (e) Arrhenius plot of contact resistances *R*_C_ *W*. Markers represent data points, dashed lines represent corresponding linear fits. b) Density of trap states *D*_tr_, (d) activation energy *E*_a_ and (f) thermionic emission barrier Φ_TE_, extracted from data in (a), (c) and (e), respectively. Labels above the bars show exact numerical values of the quantities, vertical capped lines represent the propagation of the standard error of the slope of linear fits. All values for µ_TLM_ and *R*_C_ *W* are taken at the highest common overdrive voltage across all temperatures |*V*_od_| = 1.8 V.

## 2 Results and Discussion

### 2.1 Room Temperature Characterization

The performance of OFETs can be limited by various effects, such as high contact resistance, short‐channel effects, poor quality of the gate dielectric etc.^[^ ^29^, ^43^, ^44^, ^45^ ^]^ In order for the electrical characterization of OFETs and the extracted device parameters to be reliable, devices have to exhibit a behavior which is sufficiently close to an ideal field‐effect transistor within the scope of the gradual channel approximation (GCA) (see SI, Section S6). Therefore we first analyze the characteristics of p‐ and n‐channel OFETs (C_8_‐DNTT and PhC_2_‐BQQDI, respectively) with a hydroxyl‐free (TDPA‐functionalized) Al_2_O_3_ gate dielectric measured at room temperature qualitatively. Good linearity at gate‐source voltages *V*_G_ above the threshold voltage *V*_th_ is observed for transfer characteristics in the linear regime of operation (**Figure** **1**a,e). Similarly, drain current *I*_D_ in the output characteristics closely follows an Ohmic relation at low drain‐source voltages |*V*_D_| ⩽ 1.0 V (Figure 1b,f). Furthermore, a distinct saturation regime is present at the values of *V*_D_ above the overdrive voltage *V*_od_ = *V*_G_ − *V*_th_, indicating a pinch‐off of the conduction channel.^[^ ^28^ ^]^ These are all indicators of a close‐to‐ideal field‐effect transistor behavior.^[^ ^28^ ^]^ In the case of the TLM analysis, the total resistance of the devices *R*_tot_ *W* has to scale linearly with the channel length *L* (see Equation 1).^[^ ^29^, ^46^ ^]^ As can be seen from Figure 1c,d, the total resistance extracted from the transfer characteristics does scale fairly linearly even at small channel lengths. The extracted contact resistance *R*_C_ *W* gradually decreases with *V*_od_ (Figure 1d,h); an effect attributed to a reduction of the injection barrier due to the charge carrier concentration‐dependent image forces at the interface (i.e., Schottky effect).^[^ ^31^, ^47^, ^53^ ^]^ The TLM charge carrier mobility µ_TLM_ (see Equation 1) stays fairly constant throughout most of the voltage range (see Figure 1d,h). It is worth noting that a field‐independent charge carrier mobility is also often attributed to a close‐to‐ideal OFET behavior.^[^ ^23^, ^48^ ^]^ The rest of the investigated devices behave similarly (see SI, Section S5).

After establishing the reliability of the OFET characteristics, a quantitative analysis can be performed. A compilation of the performance metrics extracted from the transfer characteristics of OFETs at room temperature is presented in **Table** **1**. Regardless of the conduction channel type, devices with a TDPA‐functionalized (hydroxyl‐free) Al_2_O_3_ gate dielectric exhibit µ_TLM_ values that exceed those of devices with a bare (hydroxylated) Al_2_O_3_ by at least one order of magnitude. Since no exposure of the devices to ambient air occurs during the full‐HV processing and characterization chain (see Section 4), this serves as a distinct indication that hydroxyl‐induced trap states on the surface of the Al_2_O_3_ gate dielectric strongly affect both hole and electron conductivity. This observation contradicts the established belief that hydroxyl groups on the surface of gate dielectrics affect primarily the n‐channel conductivity.^[^ ^23^, ^32^, ^33^ ^]^ Regarding the influence of the film morphology, we observe no correlation between the grain size in the active channel of OFETs and the charge carrier mobility (see SI, Section S3). This excludes in particular the grain boundaries as a major source of trap states at the gate dielectric‐OSC interface.^[^ ^22^, ^49^ ^]^ Thus, we can conclude that the reduced charge carrier mobility in both p‐ and n‐channel OFETs with hydroxylated gate dielectrics is caused by the hydroxyl‐induced trap states.

**Table 1:** Parameters extracted from the transfer characteristics in the linear regime of operation of OFETs measured at room temperature. For the threshold voltage *V*_th_ and the subthreshold swing *S*_sub_ average values over all devices in a TLM dataset for both forward and backward sweeps are given. For the charge carrier mobility µ_TLM_ and the contact resistance *R*_C_ *W* an average from both forward and backward sweeps at *V*_od_ = 2.2 V is taken (the highest common overdrive voltage among all studied systems at room temperature). The Schottky barrier Φ_SM_ is calculated as a difference between the work function of the source‐drain electrodes and the transport level of the OSC (see SI, Section S10).

OSC	Surface	*V*_th_ [V]	*S*_sub_ [V dec^−1^]	µ_TLM_ ^a)^ [${\mathrm{cm}}^{2}\;V^{-1}\;s^{-1}$]	*R*_C_ *W* [kΩ cm]	Φ_SM_ [eV]
PEN	TDPA	−6.7(0.5)	0.4(0.3)	0.99	6.78(0.36)	0.34(0.03)
C_8_‐DNTT	TDPA	−6.3(0.5)	0.2(0.1)	5.77	0.44(0.03)	0.61(0.05)
PhC_2_‐BQQDI	TDPA	−0.1(0.1)	0.4(0.1)	0.79	4.57(0.97)	0.65(0.04)
PEN	Al_2_O_3_	−1.3(0.4)	0.7(0.4)	0.11	207(42)	0.35(0.04)
C_8_‐DNTT^b)^	Al_2_O_3_	–	–	–	–	–
PhC_2_‐BQQDI	Al_2_O_3_	2.8(0.4)	1.2(0.2)	0.03	109(25)	0.67(0.05)

^a)^ For µ_TLM_ uncertainties are two to seven orders of magnitude lower than the actual value.

^b)^ Due to a poor performance of the devices and non‐linearity of the measured transfer characteristics a reliable analysis could not be performed for this system – see SI, Section S5.

Typically, the threshold voltage *V*_th_ is related to the concentration of trap states at the gate dielectric‐OSC interface *N*_tr_ (see SI, Section S7).^[^ ^50^, ^51^ ^]^ As expected in the case of a TDPA‐functionalized Al_2_O_3_ gate dielectric, where the surface hydroxyl groups are replaced by TDPA molecules, OFETs with a PhC_2_‐BQQDI active layer reveal a negligible threshold voltage *V*_th_ = −0.1(0.1) V (see Table 1). Both p‐channel OFETs, on the other hand, exhibit *V*_th_ ⩽ 6.3 V.^[^ ^52^ ^]^ Conversely, the subthreshold swing *S*_sub_, which is commonly associated with the density of deep trap states (see SI, Section S7),^[^ ^51^, ^53^ ^]^ shows a stronger correlation with µ_TLM_; OFETs with a TDPA‐functionalized gate dielectric exhibit an up to three times lower *S*_sub_ than those with a bare Al_2_O_3_ dielectric. The lack of correlation between *V*_th_ and µ_TLM_, and between *V*_th_ and *S*_sub_ indicates that the concentration of trap states at the gate dielectric‐OSC interface might not be the decisive factor for the mobility of the charge carriers.

The TLM analysis makes it possible to access another important performance characteristic of OFETs, namely the contact resistance *R*_C_ *W*. The contact resistance is usually described as a device effect that arises due to an inefficient charge carrier injection at the interface between the source‐drain electrodes and the OSC layer.^[^ ^29^ ^]^ Comparing values of *R*_C_ *W* for devices with different OSCs and gate dielectric treatments (see Table 1), we observe a trend similar to that of µ_TLM_ and *S*_sub_; regardless of the channel type, the contact resistance is up to two orders of magnitude lower in OFETs with a TDPA‐functionalized gate dielectric. A strong negative correlation between *R*_C_ *W* and µ_TLM_ has been recently observed in many high‐performance OFET systems and is typically attributed to various factors such as a wide depletion region at the injection interface.^[^ ^31^, ^54^, ^55^ ^]^ Notably, we also observe that *R*_C_ *W* is clearly independent of the Schottky barrier estimated by the Schottky–Mott rule, Φ_SM_ = WF − χ_OSC_ (here WF is the work function of the source‐drain electrodes measured by the Kelvin probe method (KPM) and χ_OSC_ is the energy of the OSC's transport level taken from literature – see SI, Section S10). While similar observations have been made before (see above), we are able to demonstrate that this effect is present in both p‐ and n‐channel OFETs.

### 2.2 Variable‐Temperature Characterization

The temperature sensitivity of trap states can be exploited to derive otherwise inaccessible device parameters and gain further insight into the effects of the hydroxyl‐induced trap states on the p‐ and n‐channel conductivity in OFETs. Specifically, the variable‐temperature characterization allows the extraction of the energetic density of trap states at the dielectric‐OSC interface *D*_tr_, which is derived from the temperature dependence of *V*_th_ (see **Figure** **2**a and SI, Section S7).^[^ ^50^, ^51^ ^]^ The results of the analysis reveal that *D*_tr_ is lower in devices with a TDPA‐functionalized gate dielectric regardless of the channel type (see Figure 2b): $2.5\times 10^{14}\;{\mathrm{cm}}^{-2}\;{\mathrm{eV}}^{-1}$ and $0.9\times 10^{14}\;{\mathrm{cm}}^{-2}\;{\mathrm{eV}}^{-1}$ (TDPA‐functionalized Al_2_O_3_) vs. $4.0\times 10^{14}\;{\mathrm{cm}}^{-2}\;{\mathrm{eV}}^{-1}$ and $3.7\times 10^{14}\;{\mathrm{cm}}^{-2}\;{\mathrm{eV}}^{-1}$ (bare Al_2_O_3_) for PEN and PhC_2_‐BQQDI, respectively. Notably, the difference between the density of trap states in devices with bare gate dielectrics and TDPA‐functionalized dielectrics is significantly larger for PhC_2_‐BQQDI than for PEN (77 % vs. 33 %, respectively). This indicates that TDPA‐functionalization of Al_2_O_3_ might not only eliminate the hydroxyl‐induced trap states, but also introduce additional traps, whose energy results in a preferential trapping of holes. At the same time, the difference between the densities of trap states for PEN and PhC_2_‐BQQDI OFETs with a hydroxylated Al_2_O_3_ dielectric is only 7.5 %. If the trap states induced by the surface hydroxyl groups were to affect primarily the n‐channel conductivity, one would expect the density of trap states to be much higher for PhC_2_‐BQQDI OFETs. Since this is not the case, we conclude that such trap states affect p‐ and n‐channel conductivity rather similarly. Furthermore, *D*_tr_ does not correlate with *V*_th_ derived from room temperature measurements (see Table 1), which emphasizes the necessity of variable‐temperature characterization to reliably extract OFET performance parameters.

Another charge transport characteristic accessible via the variable‐temperature analysis is the activation energy of charge carrier transport *E*_a_, which describes the average energy required to transport charge carriers between neighboring states. It is extracted from the temperature dependence of µ_TLM_, in particular in this study according to µ_TLM_∝exp (− *E*_a_/*k*_B_ *T*) (see Figure 2c,d and SI, Section S9). Devices with a TDPA‐functionalized Al_2_O_3_ gate dielectric exhibit a 4 to 5 times lower activation energy regardless of the channel type: 16.5 meV and 11.8 meV (TDPA‐functionalized Al_2_O_3_) vs. 74.3 meV and 62.6 meV (bare Al_2_O_3_) for PEN and PhC_2_‐BQQDI, respectively. Similar values have been reported in the literature before: Laudari and Guha extracted an activation energy of 76 meV for 6,13‐bis(triisopropylsilylethynyl)pentacene on a bare Al_2_O_3_ gate dielectric, approx. 3 to 4 times higher than on a hydroxyl‐free CYTOP dielectric. Similarly, the average effective charge carrier mobility µ_eff_ was around $0.03\;{\mathrm{cm}}^{2}\;V^{-1}\;s^{-1}$ and $0.18\;{\mathrm{cm}}^{2}\;V^{-1}\;s^{-1}$ for Al_2_O_3_ and CYTOP dielectrics, respectively.^[^ ^35^ ^]^ Despite different OSCs, growth processes and device structures in those reports, the results are similar to our observations not only qualitatively, but also quantitatively. Letizia et al. reported activation energies and effective charge carrier mobilities for various p‐ and n‐channel channel OFETs with a trimethylsilyl‐functionalized (hydroxyl‐free) SiO_2_ gate dielectric. They derived $\mu_{\mathrm{eff}}=0.39\;{\mathrm{cm}}^{2}\;V^{-1}\;s^{-1}$ and *E*_a_ = 30 meV for PEN,^[^ ^56^ ^]^ which agrees well with our results for PEN OFETs with a TDPA‐functionalized Al_2_O_3_. Interestingly, they also observed a relatively high threshold voltage of −10 V, similar to the threshold voltages of p‐channel OFETs reported in this study (see Table 1). Other studies have also reported low effective mobilities of p‐channel OFETs on Al_2_O_3_.^[^ ^34^ ^]^ We therefore conclude that the low charge carrier mobility in OFETs caused by hydroxyl‐induced trap states at the gate dielectric‐OSC interface is relevant both for p‐ and n‐channel conductivity. We argue that the origin of the phenomenon lies within a high activation energy of charge transport, rather than a high density of trap states, as it is often assumed. This is corroborated not only by the correlation between µ_TLM_ and *E*_a_, but also by the fact that *D*_tr_ extracted from PEN OFETs with a TDPA‐functionalized gate dielectric is only 1.6 times lower than *D*_tr_ of OFETs with a hydroxylated dielectric. In comparison, for the same material system, *E*_a_ is 4.5 times lower in devices with a hydroxylated dielectric.

It has previously been suggested that an efficient charge transport in the OSC active layer near the electrodes may be more important for reducing the contact resistance than a low Schottky (injection) barrier.^[^ ^31^ ^]^ Here we would like to make a clear distinction between the Schottky barrier estimated using the Schottky–Mott rule (Schottky–Mott barrier, denoted Φ_SM_; see Subsection 2.1) and the Schottky barrier extracted using the thermionic emission theory (thermionic emission barrier, denoted Φ_TE_; see SI, Section S9). In the case of metal‐OSC interfaces the Schottky–Mott rule is generally not valid, since phenomena such as partial and integer charge transfer, interfacial dipole, “cushion” effect, Fermi level pinning etc., define the energy level alignment at the interface.^[^ ^57^, ^58^, ^59^ ^]^ This might explain why no correlation between the values of the theoretical Schottky‐Mott barrier Φ_SM_ and the actual room‐temperature contact resistance *R*_C_ *W* is observed. Considering the strong negative correlation between *R*_C_ *W* and µ_TLM_, discussed in Subsection 2.1, we could assume that the charge carrier mobility plays a more important role in reducing the contact resistance. However, we are not limited to the Schottky–Mott rule in the estimation of the Schottky barrier at the electrode‐OSC interface. Specifically, the variable‐temperature TLM analysis allows extraction of the thermionic emission barrier directly from the temperature dependence of the contact resistance (see Figure 2e,f and SI, Section S9).^[^ ^53^ ^]^ We find that the values of Φ_TE_ are 3 to 4 times lower in devices with a TDPA‐functionalized gate dielectric: 31.7 meV and 17.1 meV (TDPA‐functionalized Al_2_O_3_) vs. 98.8 meV and 71.5 meV (bare Al_2_O_3_) for PEN and PhC_2_‐BQQDI, respectively. This agrees well with the trend observed for the contact resistance extracted at room temperature and indicates that the thermionic emission barrier plays a significant role in reducing the contact resistance in OFETs. Interestingly, the values of Φ_TE_ and *E*_a_ exhibit a strong positive correlation. This indicates a connection between the thermionic emission barrier and the efficiency of the charge carrier transport in the conduction channel. Such connection might be explained by the dependence of the injection current on the charge carrier density and transport efficiency near the electrode, for example due to the Schottky effect, as has been suggested before.^[^ ^31^, ^60^, ^61^, ^62^, ^63^ ^]^ Specifically, our findings demonstrate that a lower activation energy of charge transport yields a lower thermionic emission barrier. While identifying the exact nature of this effect is beyond the scope of this study, this knowledge might prove valuable in finding new approaches to optimizing OFET performance.

## 3 Conclusion

In this study we report a full‐HV variable‐temperature TLM analysis of p‐ and n‐channel thin‐film OFETs, in which devices with bare (hydroxylated) Al_2_O_3_ and TDPA‐functionalized (hydroxyl‐free) Al_2_O_3_ gate dielectrics are compared. Regardless of the channel type, the room temperature analysis yields a significantly lower charge carrier mobility in devices with a hydroxylated dielectric. This leads to the conclusion that the decrease in the charge carrier mobility of OFETs with a bare Al_2_O_3_ gate dielectric is caused solely by the trap states originating from the hydroxyl groups on the surface of the dielectric. These findings contradict the common belief of the hydroxyl‐induced trap states affecting primarily n‐channel conductivity. The nature of the effect can be explained using the variable‐temperature TLM analysis. In particular, the charge carrier mobility of devices with a hydroxylated dielectric is limited mostly by a high activation energy of the charge carrier transport, rather than by the density of trap states at the gate dielectric‐OSC interface. Furthermore, we demonstrate that the contact resistance exhibits a positive correlation with the Schottky barrier extracted using the thermionic emission theory, while being independent of the Schottky barrier estimated by the Schottky–Mott rule. The thermionic emission barrier is linked to the efficiency of the charge transport in the active channel via the activation energy of charge transport. This observation once again stresses the importance of a high‐quality gate dielectric for low contact resistance in OFETs. While the variable‐temperature analysis approach presented in this study requires a tedious HV processing and characterization chain that is not scalable for commercial purposes, the acquired results make it possible to identify key factors that limit the performance of both p‐ and n‐channel OFETs. These findings shed light on the peculiarities of the charge transport in organic electronic devices and will ultimately benefit the development of more efficient devices, thus opening up possibilities for further improvements in organic electronics.

## 4 Experimental Section

### Substrate Preparation

BGBC OFET substrates used in this study comprise of the following elements: A highly p‐doped silicon wafer as a substrate; a layer of thermally evaporated aluminum as a global gate electrode; a layer of Al_2_O_3_ with a nominal thickness of 20 nm as a gate dielectric (grown via atomic layer deposition) and gold (Au) source‐drain electrodes, thermally evaporated through a patterning mask at room temperature to a thickness of 30 nm to 40 nm. The designed channel length *L* varies from 2 µm to 100 µm; the designed channel width is either 200 or 500 µm. Functionalization of the dielectric surface is realized by submerging the substrate into a TDPA solution in isopropyl alcohol prior to source‐drain electrode deposition.^[^ ^45^ ^]^ For OFETs that require a bare Al_2_O_3_ dielectric, the substrates are subjected to a radio‐frequency O_2_ plasma after the deposition of the source‐drain electrodes. See SI, Section S1 for further details.

### OMBD

Substrates are brought into an ultra‐high vacuum (UHV) OMBD system (base pressure of 2 × 10^−9^ mbar) and are annealed at 450 K for 5 min. According to various experimental studies, at this temperature more than a half of the hydroxyl groups on an Al_2_O_3_ surface remain intact.^[^ ^64^, ^65^, ^66^ ^]^ Upon reaching the room temperature, the work function of Au on the substrate is measured via Kelvin probe method. OMBD is performed from a Knudsen evaporation cell at a substrate temperature of 300, 342, and 450 K for PEN, C_8_‐DNTT and PhC_2_‐BQQDI, respectively, and a deposition rate of 3 Å min^−1^ to 6 Å min^−1^ to a nominal film thickness of 20 nm. The deposition rate is monitored with a quartz crystal microbalance sensor. Deposition is performed through a shadow mask that allows separation of the devices and a significant reduction of the crosstalk between the source‐drain and gate electrodes. The OSCs were acquired from the following sources and used as is: PEN from *Sigma‐Aldrich* (purity >99.9 %) C_8_‐DNTT synthesized by *Martina Volpi* in the group of *Prof. Dr. Yves Geerts* (*Laboratory of Polymer Chemistry*, *Université Libre de Bruxelles*), PhC_2_‐BQQDI from *FUJIFILM Wako Pure Chemical Corporation* (purity >99.0 %). See SI, Section S2 for further details.

### Electrical Characterization

OFETs are transferred under static vacuum to an UHV electrical characterization chamber (base pressure of 9 × 10^−8^ mbar).^[^ ^40^ ^]^ Electrical characterization is performed using a *Hewlett‐Packard HP4145B* parameter analyzer controlled externally via *SweepMe!*.^[^ ^67^ ^]^ Devices are contacted with two independently‐controlled *Kleindiek MMA3* piezo‐actuated micromanipulators equipped with *Low Current Measurement Kits* and *Mercia Semiconductor 7B‐25G* gold‐coated tungsten probes with a tip radius of 25 µm. The source electrode is connected to the common ground of the parameter analyzer, the drain and the gate electrodes are connected to the source‐measurement units. Electrical characterization is performed in the dark with a series of up to 5 fast forward‐backward transfer sweeps before measuring each individual OFET to eliminate photogenerated charge carriers.^[^ ^68^ ^]^ Control of specimen temperature is realized via a combination of resistive heating and liquid nitrogen cooling. The specimen temperature is monitored constantly via an electrically insulated K‐type thermocouple mounted on the specimen holder. The variable‐temperature analysis is performed at 6 to 7 temperatures in the range from 120 to 403 K, in the order from low to high temperatures (without repeating the low‐temperature measurements). Significant changes in the structure of thin films due to prolonged exposure to elevated temperatures is not expected in this temperature range. Thus the temperature‐induced aging effects are assumed to be negligible – see SI, Section S4 for more details.

### Data Analysis

Analysis of the electrical characteristics of OFETs is performed within the scope of the GCA (see SI, Section S6). Threshold voltage *V*_th_ is extracted from the linear part of the transfer characteristics in the linear regime of operation as the intercept of the linear fit with *I*_D_ = 0 A (SI, Equation 6.3).^[^ ^28^ ^]^ Subthreshold swing *S*_sub_ is defined as an inverse slope of log_10_|*I*_D_| in the onset range of the transfer characteristics in the linear regime of operation (SI, Equation 7.3).^[^ ^53^ ^]^ Density of trap states *D*_tr_ is derived from the temperature dependence of the threshold voltage *V*_th_ (SI, Equation 7.2).^[^ ^50^, ^51^ ^]^ Width‐normalized total resistance of an OFET *R*_tot_ *W* is defined as:^[^ ^29^, ^31^ ^]^

(1) $$R_{\mathrm{tot}}W\left(L\right)=\frac{L}{\mu_{\mathrm{TLM}}C_{\mathrm{diel}}V_{\mathrm{od}}}+R_{C}W,$$

where *W* is the channel width, *L* is the factual channel length measured via optical microscopy,^[^ ^69^ ^]^ *R*_C_ *W* is the width‐normalized contact resistance, µ_TLM_ is the charge carrier mobility in the conduction channel, *C*_diel_ is the aerial capacitance of the gate dielectric (0.43 µF cm^−2^ and 0.30 µF cm^−2^ for bare and TDPA‐functionalized Al_2_O_3_ dielectric, respectively; see SI, Equation 6.4) and *V*_od_ is the overdrive voltage, *V*_od_ = *V*_G_ − *V*_th_. Extraction of *R*_C_ *W* and µ_TLM_ is performed by plotting the total resistance of devices with varying *L* at a predefined *V*_od_ and fitting the data with a linear function. It follows from Equation 1 that at *L* → 0 m, *R*_tot_ *W* → *R*_C_ *W*, and thus the contact resistance is extracted as an extrapolation of the fitted function at *L* = 0 m. µ_TLM_ is extracted from the slope of the fit in the first term on the right side of Equation 1. The TLM analysis is performed using the transfer characteristics in the linear regime of operation, i. e., *R*_tot_ *W*(*V*_od_) = *V*_D_/*I*_D_(*V*_od_). See SI, Section S8 for further details.

## Conflict of Interest

The authors declare no conflict of interest.

## Supporting information

Supporting Information

## Data Availability

The data that support the findings of this study are available from the corresponding author upon reasonable request.
