# Supplementary material for: Unveiling the Effects of Hydroxyl‐Induced Trap States on the Charge Transport in p‐ and n‐Channel Organic Field‐Effect Transistors through Variable‐Temperature Characterization
Source: Adv Mater. 2025 Jun 12;37(34):2505631. doi: 10.1002/adma.202505631 (PMC12392864; doi:10.1002/adma.202505631)
Supplement: Supplementary file 1 — Supporting Information [file ADMA-37-2505631-s001.pdf]

ADVANCED MATERIALS

Supporting Information

for *Adv. Mater.*, DOI 10.1002/adma.202505631

Unveiling the Effects of Hydroxyl-Induced Trap States on the Charge Transport in p- and n-Channel Organic Field-Effect Transistors through Variable-Temperature Characterization

Yurii Radiev, Tobias Wollandt, Hagen Klauk and Gregor Witte**

Supporting Information

Unveiling the Effects of Hydroxyl-Induced Trap States on the Charge Transport in p- and n-Channel Organic Field-Effect Transistors through Variable-Temperature Characterization

Yurii Radiev[1] Tobias Wollandt[2] Hagen Klauk[2] Gregor Witte[1]

April 27, 2025

Yurii Radiev (ORCID 0000-0001-6969-546X), Gregor Witte (ORCID 0000-0003-2237-0953)
Email: yurii.radiev@physik.uni-marburg.de, gregor.witte@physik.uni-marburg.de
[1]Philipps-Universität Marburg, Renthof 7, 35032 Marburg, Germany
Tobias Wollandt (ORCID 0000-0002-0843-4159), Hagen Klauk (ORCID 0000-0003-4563-5635)
[2]Max Planck Institute for Solid State Research, Heisenbergstr. 1, 70569 Stuttgart, Germany

Contents

1 Substrate Preparation **3**

2 OFET Preparation **4**

3 Morphology of Thin Films **5**

4 Electrical Characterization **5**

5 Compilation of OFET Characteristics **7**

6 Gradual Channel Approximation **9**

7 Density of Trap States **9**

8 Transfer Length Method **10**

9 Arrhenius Analysis **11**

10 Tabular Data **12**

List of Figures

S1 Effects of O_2 plasma treatment on Al_2O_3 gate dielectric 4
S2 AFM micrographs of thin films 5
S3 Full HV process chain 6
S4 Transfer characteristics of OFETs measured at room temperature 8

List of Tables

S1 Detailed statistics of room-temperature OFET performance parameters 12

List of Acronyms

AFM atomic force microscopy 1, 3, 4, 5
Al aluminum 4, 5
Al_2O_3 aluminum oxide 1, 2, 3, 4, 5, 7, 8, 9, 12
ALD atomic layer deposition 3, 4
Au gold 4
BGBC bottom gate-bottom contact 3
C_8-DNTT 2,9-dioctylnaphtho[2,3-b]naphtha[2',3':4,5]thieno[2,3-d]thiophene 4, 8, 12
DNTT dinaphtho[2,3-b:2',3'-f]thieno[3,2-b]thiophene 6, 7
DOS density of states 10
GCA gradual channel approximation 9
HV high vacuum 1, 6, 7
KPM Kelvin probe method 4
LED light-emitting diode 6
LN_2 liquid nitrogen 5
O_2 molecular oxygen 1, 3, 4
OFET organic field-effect transistor 1, 2, 3, 4, 6, 7, 8, 9, 12
OMBD organic molecular-beam deposition 4, 6
OSC organic semiconductor 3, 4, 6, 9, 10, 11, 12
PEN pentacene 4, 5, 6, 7, 12
PhC_2-BQQDI phenylalkyl-substituted 3,4,9,10-benzo[*de*]isoquinolino[1,8-*gh*]quinolinetetracarboxylic diimide 4, 5, 6, 7, 12
PTFE polytetrafluoroethylene 5
QCM quartz crystal microbalance 4
RF radio-frequency 3, 4
Si silicon 4
SiO_2 silicon dioxide 3
SMU source-measurement unit 5
TDPA tetradecylphosphonic acid 3, 4, 5, 7, 9, 12
TLM transfer length method 3, 6, 7, 8, 10, 11, 12
UHV ultra-high vacuum 4

List of Symbols

V_{D} drain voltage 6, 9
V_{G} gate voltage 6, 9
I_{D} drain current 9
W channel width 9
L channel length 9
μ_{eff} effective charge carrier mobility 9
C_{diel} aerial capacitance of the gate dielectric 9
V_{th} threshold voltage 9
ϵ_0 vacuum permittivity 9
$\epsilon_{\mathrm{Al_2O_3}}$ dielectric constant of aluminum oxide 9

$d_{Al_2O_3}$ thickness of the aluminum oxide gate dielectric layer 9
d_{TDPA} thickness of the tetradecylphosphonic acid dielectric layer 9
ϵ_{TDPA} dielectric constant of tetradecylphosphonic acid 9
N_{tr} aerial concentration of trap states 9
T temperature 9
V_{onset} onset voltage 9
q elementary charge 9
D_{tr} energetic density of trap states 9
k_B Boltzmann constant 9
$R_{tot}W$ width-normalized total resistance 10
R_S interfacial resistance at the source electrode 10
R_{ch} resistance of the active channel 10
R_D interfacial resistance at the drain electrode 10
R_CW width-normalized contact resistance 10, 11
μ_{TLM} charge carrier mobility extracted via transfer length method 10
V_{od} overdrive voltage 10
Φ_{TE} injection barrier within the thermionic emission model 11
E_a activation energy of charge carrier transport 11
A^* Richardson constant 11
Φ_{SM} Schottky barrier estimated by the Schottky-Mott rule 12

1 Substrate Preparation

The bottom gate-bottom contact (BGBC) organic field-effect transistors (OFETs) is chosen because it allows to avoid any intercalation of electrode and/or dielectric material into the organic semiconductor (OSC), which occurs in top gate and top contact structures.[1–4] Thus sharp interfaces with the active layer are ensured. The gold source-drain electrodes are thermally evaporated from a molybdenum-coated tungsten boat in a vacuum evaporation setup with a base pressure of 1×10^{-8} mbar. Gold nuggets with a purity of 99.99 % purchased from *Agosi AG* are used A laser-cut *Kapton* (polyimid) shadow mask from *CADiLAC Laser GmbH* with a thickness of 50 µm or a stencil silicon dioxide (SiO_2) mask is used for electrode patterning. The substrate is kept at room temperature during the electrode deposition. Specimens that require a bare aluminum oxide (Al_2O_3) surface are subjected to a radio-frequency (RF) molecular oxygen (O_2) plasma in a *Harrick Vacuum Basic Plasma Cleaner PDC-32G-2.* The plasma cleaning is performed at room temperature for a duration of 5 min at an RF power of 18 W and an O_2 pressure of 0.8 mbar. To confirm that tetradecylphosphonic acid (TDPA) is removed from the surface after O_2 plasma treatment, a series of atomic force microscopy (AFM) and contact angle measurements was performed. Figure S1 (a) demonstrates an AFM topography micrograph of a TDPA-functionalized Al_2O_3 gate dielectric used in this study. The micrograph reveals irregularities (light regions on the micrograph) which are not typical for an Al_2O_3 surface grown by atomic layer deposition (ALD) and arise from the TDPA functionalization. The surface is clearly hydrophobic with a contact angle $\alpha = 110(2)^\circ$ (distilled water in air, droplet volume of 2 µL, measured within 30 s after droplet deposition, presented value is a mean of measurements from five droplets) – a clear indication of a successful functionalization of a natively hydrophilic Al_2O_3 surface, see inset in Figure S1 (a).[5, 6] After the measurements the specimen was subjected to the O_2 plasma treatment as described above. After the O_2 plasma treatment the surface exhibits much higher homogeneity than before, as demonstrated in Figure S1 (b). Furthermore, the surface is highly hydrophobic, with $\alpha < 1^\circ$, indicative of a bare Al_2O_3 surface. The root mean squared areal surface roughness S_{RMS} drops by at least 36 % after the O_2 plasma treatment, from 0.87 nm to 0.55 nm before and after the treatment, respectively (measured across the whole images in Figure S1). An increase of the thickness of the gate dielectric can be excluded: Since

it is grown by an ALD on a silicon substrate, there is no source of aluminum to form additional layers of the oxide. As to the quality of the surface, from a study by Geiger et al. it follows that S_{RMS} of Al_2O_3 dielectric is almost identical to that of the underlying substrate,[7] especially at low values of $S_{RMS} < 2\,nm$. Thus a low S_{RMS} of the Al_2O_3 dielectric after the O_2 plasma treatment indicates a high quality of the surface (particularly considering that locally, i. e. on patch with an area of $1\,\mu m^2$, the surface roughness is only $S_{RMS} = 0.24\,nm$).

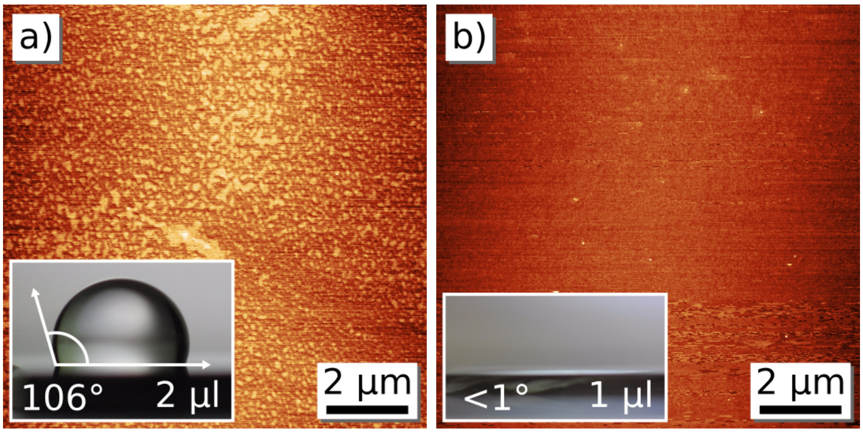

Figure S1: AFM topography micrographs of a TDPA-functionalized Al_2O_3 gate dielectric (a) and the same specimen after being subjected to an O_2 plasma treatment for 5 min at a RF power of 18 W and a chamber pressure of 0.8 mbar (b). Data processing consisted of a mean plane subtraction and the alignment of rows using the method of medians.[8, 9] In both micrographs the colormap is set to a range from 0 nm to 6.9 nm (from darker to brighter, respectively) to make a direct comparison between the micrographs possible. Photographs in the insets demonstrate water droplets used for contact angle measurements on the corresponding surfaces.

2 OFET Preparation

The organic molecular-beam deposition (OMBD) and Kelvin probe method (KPM) system is comprised of two separate ultra-high vacuum (UHV) chambers with a transfer system. The pre-deposition annealing is performed in the OMBD chamber with a base pressure of 2×10^{-9} mbar. Specimens are allowed to cool down to room temperature in the interconnected KPM chamber (base pressure of 2×10^{-10} mbar), where the work function of gold is measured on up to four specifically designated grounded gold pads.[10] The measurement is performed using a *Besocke Delta Phi Kelvin Probe S* with a gold (Au) electrode referenced on a thoroughly prepared Au(111) surface (Fermi level $E_F = 5.33\,eV$).[11] Afterwards, the OSCs are deposited in the OMBD chamber from a Knudsen evaporation cell at the temperature of 472 K, 558 K and 633 K for pentacene (PEN), 2,9-dioctylnaphtho[2,3-b]naphtha[2',3':4,5]-thieno[2,3-d]thiophene (C_8-DNTT) and phenylalkyl-substituted 3,4,9,10-benzo[*de*]isoquinolino-[1,8-*gh*]quinolinetetracarboxylic diimide (PhC_2-BQQDI), respectively. The deposition is started only after a stabilization of the deposition rate, which is measured with a quartz crystal microbalance sensor. The deposition rate is also measured at the end of the deposition to ensure its consistency. Deposition is performed through an aluminum shadow mask that aligns with the patterned source-drain electrodes (see Figure S3) by means of precisely machined alignment pins. The shadow mask is stored inside the load-lock chamber (base pressure of 1.6×10^{-8} mbar), where it is placed onto the specimen holder before the OMBD. After the OSC is deposited, the shadow mask is removed to allow access to the source-drain electrodes for selective electrical characterization of OFETs (see Section 4).

3 Morphology of Thin Films

AFM imaging was performed under ambient conditions with an *Agilent Technologies PicoPlus Atomic Force Microscope Series 5500* in a tapping mode of operation using *MikroMasch HQ NSC15/Al BS Standard Tapping Mode AFM Probes*. As can be seen from Figure S2, no significant change in the film morphology was observed for films deposited on a TDPA-functionalized Al_2O_3 gate dielectric. Furthermore, one can see that TDPA-functionalization of the dielectric results in different effects for PEN and PhC_2-BQQDI, namely grain size decrease and increase, respectively. Thus there is no consistency with the reported threshold voltage change.

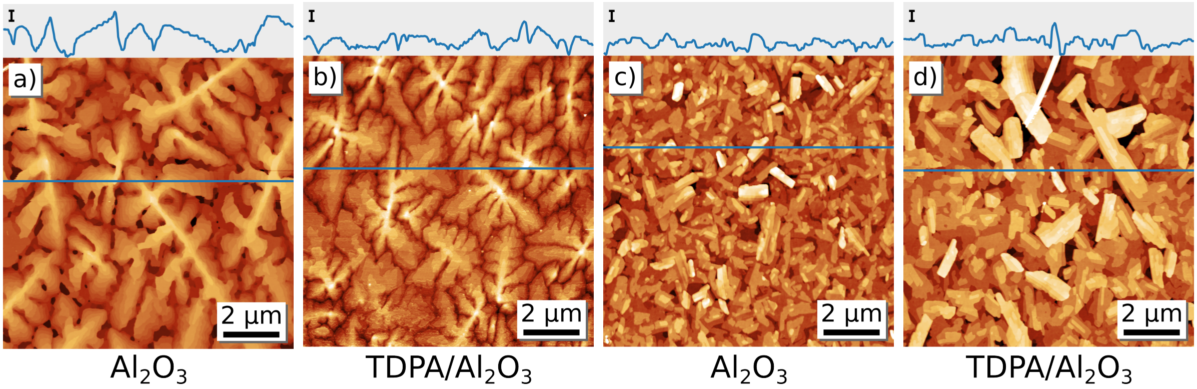

Figure S2: AFM topography micrographs of thin films of PEN (a-b) and PhC_2-BQQDI (c-d). Films grown on bare Al_2O_3 dielectric are shown in panels (a) and (c), films grown on TDPA-functionalized dielectric are shown in panels (b) and (d). The deposition temperatures were 300 K and 450 K for PEN and PhC_2-BQQDI, respectively. Data processing consisted of a mean plane subtraction and the alignment of rows using the method of median of differences.[8, 9]. Blue traces above the micrographs are height profiles along the x-axis (fast axis) of the scan (the location of the profile is marked by a blue horizontal line the micrograph). Vertical scalebar in each height profile corresponds to 5 nm.

4 Electrical Characterization

After the active layer deposition, specimens are allowed to cool down to room temperature and are then transferred under a static vacuum (8×10^{-7} mbar) to the electrical characterization chamber (base pressure of 9×10^{-8} mbar).[12] In the chamber the stainless steel specimen holder is placed onto a copper stage, with the top plate of the holder securely fixed against the guide rails of the stage via a spring-loaded mechanism. The copper stage is connected to one of the source-measurement units (SMUs) of the *Hewlett-Packard HP4145B* parameter analyzer and serves as a gate electrode. An electrically insulated resistive heater is built into the stage which allows heating up the specimen. Cooling down is performed using a thermally conductive connection to a copper heatsink, through which a controlled flow of liquid nitrogen is circulated. Thermal conductivity and electrical insulation between the stage and the heatsink is ensured by using sapphire washers on all physical connections. The stage itself is mounted on an aluminum plate using polytetrafluoroethylene washers to ensure electrical and heat insulation. Two *Kleindiek MMA3* piezo-actuated micromanipulators with *Low Current Measurement Kit* probe tip holders are also mounted on the same plate. The micromanipulators allow selective targeting of devices' source and drain electrodes via a remote controller. The drain electrode is connected to one of the SMUs of the parameter analyzer, while the source electrode is connected to the common ground of the instrument. The electric probe tips used are *Mercia Semiconductor 7B-25G* gold-coated tungsten probes with a tip radius of 25 µm. Probe positioning is monitored via a top-view

camera and an angled-view camera, which allows precise positioning of the probe tips without applying an excessive vertical force.

Electrical characterization is performed in the dark with specimens being exposed to a generic white light-emitting diode light only for a short period of time between the measurements to reposition the electrical probes. To eliminate the effect of the photogenerated charge carriers, a series of up to 5 fast forward-backward transfer sweeps is performed before each measurement.[13] The transfer characteristics in the linear regime of operation are measured at a drain voltage V_D of either 0.1 V or 1.0 V, with a typical absolute maximum gate voltage $|V_\mathrm{G}| = 10.0$ V and a typical gate voltage range of 10 V. Every transfer curve contains at least 80 measurement points, as per the *IEEE Standard*.[14] The specimen temperature is monitored constantly via an electrically insulated K-type thermocouple mounted at the specimen holder. In the variable-temperature analysis, the electrical characterization is performed at 6 to 7 different temperatures in the range from 120 K to 403 K (from low to high temperatures, without repeating the low-temperature measurements).

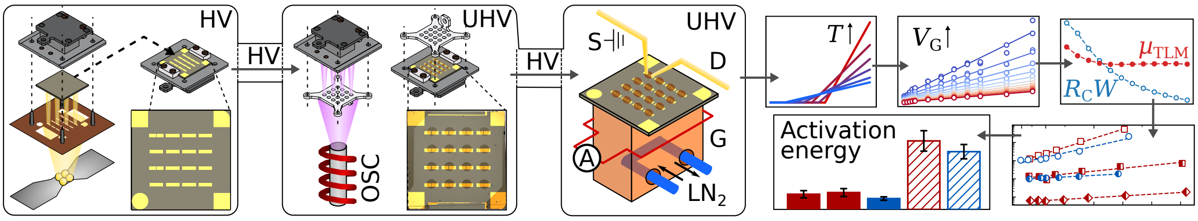

Figure S3: Full HV process chain, starting left with the electrode deposition, followed by a vacuum transfer into the OMBD chamber, where the deposition of the OSC layer is performed, followed by a vacuum transfer into the electrical characterization chamber, where temperature dependent transfer characteristics are measured for devices with varying channel length L. The transfer characteristics are then used for the TLM analysis. The charge carrier mobility μ_TLM and the contact resistance $R_\mathrm{C}W$ are extracted from TLM analysis at different temperatures, which allows to perform the Arrhenius analysis and extract the activation energy of the charge transport E_a and the Φ_SM, respectively. The extracted values are compared between specimens with different functionalization of the gate dielectric.

We argue that the bias-stress effects in the case of this study are negligible, since typically they occur if the drain current is applied continuously on a timescale of multiple hours to days, with the device recovery on a timescale of tens of minutes.[15] In in this study, however, the transfer characteristics of a single device are measured within 3-5 minutes, followed by a time period of at least 1-2 hours required to measure other devices on the substrate, and to reach and equilibrate the substrate temperature. Thus not only the period of a continuous application of the drain voltage is short, but in comparison to it the recovery period is also much larger.

As for the aging effects, in our previous study on PEN OFETs we have shown that in the first few days of vacuum storage there are no significant changes in the current-voltage characteristics of devices.[12] Furthermore, dinaphtho[2,3-b:2',3'-f]thieno[3,2-b]thiophene (DNTT) OFETs (and OFETs based on its C_n- derivatives) are known to be rather stable even when stored under ambient conditions for several months.[16, 17] Similar is true for PhC$_2$-BQQDI OFETs, with no significant changes in the device performance after being stored in air for more than 4 months.[18] We are also aware that thermal annealing might introduce structural changes in the films and thus influence the device performance. For example, in our previous studies we have shown that ultra-thin films (up to 2.5 nm nominal thickness) of DNTT dewet even at room temperature, significantly impacting the device performance after only 4 hours of storage.[19] This was not the case for the films with a nominal thickness of 25 nm, where the devices retained their performance after 20 days of storage, consistent with other studies on the aging effects of DNTT films.[19] Thus we can exclude structural changes due to dewetting for our films with a nominal

thickness of 20 nm. We also argue that the structural changes due to heating would be negligible for the temperature range used for the variable-temperature analysis in this study. Indeed, PEN-based OFETs are known to show no noticeable changes in performance upon annealing at temperatures of up to 410 K.[20] Similarly, PhC_2-BQQDI-based OFETs show no significant changes in performance when annealed at temperatures of up to 453 K.[18] OFETs based on DNTT (and its C_{10}- derivative) are more susceptible to thermal annealing (at temperatures of up to 423 K), however the effect can still be considered rather small, especially for C_{10}-DNTT.[21] It is worth noting, however, that the annealing experiments with OFETs are typically performed either under ambient (Ref.[20]) or protective gas atmosphere (Refs.[18, 21]), and not in high vacuum (HV). Therefore, one could argue that the observed annealing effects might be caused not only by the structural changes in the film, but also by a higher reactivity of the film with the surrounding atmosphere. Thus we expect that due to the HV electrical characterization and low temperatures used for the variable-temperature analysis implemented in this study, any changes in the device performance due to heating will be negligible.

5 Compilation of OFET Characteristics

Room-temperature OFET transfer characteristics in the linear regime of operation of all devices investigated in this study (120 devices in total) are given in Figure S4. Note that in panels (d) and (f) (OFETs with a bare Al_2O_3 gate dielectric) the y-axis scale ($R_{\mathrm{tot}}W$) of the transfer length method (TLM) plots (center) has a 1×10^3 multiplier, meaning that the total resistance at low overdrive voltages is in the $\mathrm{M}\Omega \times \mathrm{cm}$ range. The p-channel devices with a TDPA-treated gate dielectric demonstrate a small, but still non-negligible hysteresis (see Table S1). We note that this might be caused by the hydroxyl groups which remain on the surface after the functionalization. Another cause, as discussed in Subsection 2.2 of the main text, could be that TDPA itself introduces shallow traps that can be populated/depopulated during the measurement. A similar phenomenon has been previously observed for OFETs with functionalized gate dielectrics, with the magnitude of the hysteresis being dependent on the chemical structure of the molecule used for functionalization.[5] Finally, some of the photogenerated charge carriers might remain trapped even after the initial "fast" sweeps performed before the actual measurement. Such trapped charge carriers are known to cause a threshold voltage shift in DNTT OFETs,[22] which could contribute to the observed ΔV_{th} in p-channel devices with a TDPA-functionalized gate dielectric.

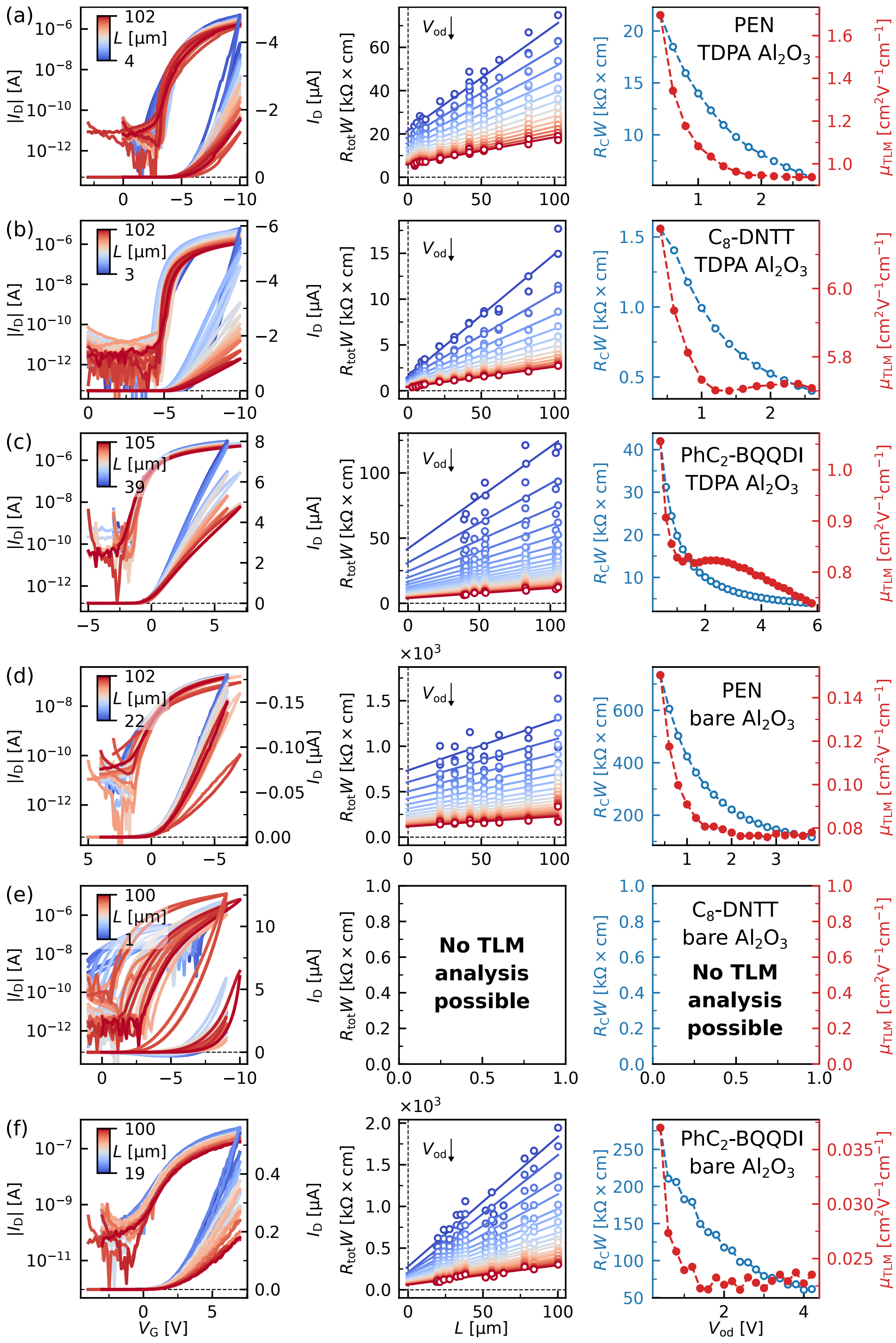

Figure S4: Transfer characteristics in the linear regime of operation of the OFETs investigated in this study measured at room temperature (left), corresponding TLM plots (center), extracted overdrive voltage-dependent contact resistances $R_C W$ and charge carrier mobilities μ_{TLM} (right). Note the poor quality of transfer characteristics for the C_8-DNTT OFETs with a bare Al_2O_3 dielectric in panel (e).

6 Gradual Channel Approximation

Within the scope of the gradual channel approximation (GCA) for an OFET operating in a linear regime, the drain current I_{D} can be written as:[4]

$$I_{\mathrm{D}} = \frac{W}{L}\mu_{\mathrm{eff}}C_{\mathrm{diel}}\left[\left(V_{\mathrm{G}} - V_{\mathrm{th}}\right)V_{\mathrm{D}} - \frac{1}{2}V_{\mathrm{D}}^{2}\right]. \tag{6.1}$$

Here I_{D} is the measured drain current, W is the width of the device channel, L is the length of the device channel, μ_{eff} is the effective charge carrier mobility, C_{diel} is the areal capacitance of the dielectric layer, V_{G} is the applied gate voltage, V_{th} is the threshold voltage and V_{D} is the applied drain voltage. By definition of the linear regime of operation, $|V_{\mathrm{D}}| << |V_{\mathrm{G}}|$, and thus the term inside the brackets in Equation 6.1 can be reduced to $\left(V_{\mathrm{G}} - V_{\mathrm{th}}\right)V_{\mathrm{D}}$. We can then rewrite Equation 6.1 as:

$$I_{\mathrm{D}} = \frac{W}{L}\mu_{\mathrm{eff}}C_{\mathrm{diel}}\left(V_{\mathrm{G}} - V_{\mathrm{th}}\right)V_{\mathrm{D}}. \tag{6.2}$$

Since $I_{\mathrm{D}} \propto V_{\mathrm{G}}$, the effective charge carrier mobility can be derived from the slope of the linear fit of the linear part of the curve according to Equation 6.2:

$$\mu_{\mathrm{eff}} = \frac{L}{WC_{\mathrm{diel}}V_{\mathrm{D}}}\frac{\partial I_{\mathrm{D}}}{\partial V_{\mathrm{G}}}, \tag{6.3}$$

with the threshold voltage V_{th} defined as an intercept of the extrapolated fitted curve with x-axis. For devices presented in this study, dielectric capacitance C_{diel} is defined as:

$$C_{\mathrm{diel}} = \frac{\epsilon_0\epsilon_{\mathrm{Al_2O_3}}}{d_{\mathrm{Al_2O_3}}} = 4.3\,\mathrm{mF\,m^{-2}} = 0.43\,\mathrm{\mu F\,cm^{-2}}, \tag{6.4a}$$

$$C_{\mathrm{diel}} = \frac{\epsilon_0}{\frac{d_{\mathrm{Al_2O_3}}}{\epsilon_{\mathrm{Al_2O_3}}} + \frac{d_{\mathrm{TDPA}}}{\epsilon_{\mathrm{TDPA}}}} = 3.0\,\mathrm{mF\,m^{-2}} = 0.3\,\mathrm{\mu F\,cm^{-2}}. \tag{6.4b}$$

Here ϵ_0 is the vacuum permittivity; $\epsilon_{\mathrm{Al_2O_3}}$ is the dielectric constant of Al_2O_3; $d_{\mathrm{Al_2O_3}}$ is the thickness of the Al_2O_3 dielectric layer; d_{TDPA} is the thickness of a TDPA monolayer on Al_2O_3; ϵ_{TDPA} is the dielectric constant of TDPA. Equation 6.4a is used in the case of a bare Al_2O_3 gate dielectric, Equation 6.4b is used in the case of a TDPA-functionalized Al_2O_3 gate dielectric.

7 Density of Trap States

The concentration of trap states at the dielectric-OSC interface N_{tr} at a certain measurement temperature T can be estimated using the following expression:

$$N_{\mathrm{tr}} \approx C_{\mathrm{diel}}V_{\mathrm{th}}/q \tag{7.1}$$

under the assumption $V_{\mathrm{onset}} \approx 0\,\mathrm{V}$;[23] here q is the elementary charge. The density of trap states close to the transport level D_{tr} can be extracted from the temperature dependence of the threshold voltage in Equation 7.1 under an assumption of quasi-linearity of $V_{\mathrm{th}}(T)$:[23–25]

$$D_{\mathrm{tr}} = \frac{\partial N_{\mathrm{tr}}(T)}{\partial E} = \left(\frac{C_{\mathrm{diel}}}{k_{\mathrm{B}}q}\right)\frac{\partial V_{\mathrm{th}}}{\partial T}, \tag{7.2}$$

where k_{B} is the Boltzmann constant. The subthreshold swing S_{sub} extracted from the transfer characteristics in the linear regime has a following dependence on N_{tr} and temperature T:[4, 23, 25, 26]

$$S_{\text{sub}} = \frac{k_{\text{B}} T ln(10)}{q} \left(\frac{q^2 N_{\text{tr}}}{C_{\text{diel}}} + 1 \right), \tag{7.3}$$

from which the lowest theoretically possible subthreshold swing at room temperature ($T = 300\,\text{K}$) can be derived at a limit of $N_{\text{tr}} \to 0$:

$$S_{\text{sub}} = \frac{k_{\text{B}} T ln(10)}{q} \approx 60\,\text{mV}\,\text{dec}^{-1}. \tag{7.4}$$

Application of Equation 7.3 is limited to measurements with a sufficiently high on-off ratio, where S_{sub} can be extracted reliably.[23]

It is worth mentioning that numerous methods (both analytical and computational) for extracting a trap density of states (DOS) have been reported in the literature.[27–35] However, a conclusive picture has not yet been obtained: Depending on the method of choice and even the initial parameters within the same method, the results change drastically.[34, 35] Thus in this report no attempt at extracting the trap DOS is made.

8 Transfer Length Method

The TLM is based on an assumption that the total width-normalized resistance $R_{\text{tot}}W$ of a device can be described by an equivalent circuit of three resistances connected in series: source resistance R_{S}, channel resistance R_{ch} and drain resistance R_{D}:[36]

$$R_{\text{tot}}W = R_{\text{ch}} + R_{\text{S}} + R_{\text{D}}. \tag{8.1}$$

R_{S} and R_{D} are considered to be channel length-independent and arise due to a voltage drop at the OSC-electrode interface. The TLM analysis does not distinguish between R_{S} and R_{D} thus only allows to extract a combined resistance, also known as contact resistance $R_{\text{C}}W = R_{\text{S}} + R_{\text{D}}$. $R_{\text{tot}}W$ thus only depends on the channel length via R_{ch} and Equation 8.1 can be rewritten as:

$$R_{\text{tot}}W(L) = R_{\text{ch}}(L) + R_{\text{C}}W = \frac{L}{\mu_{\text{TLM}} C_{\text{diel}} V_{\text{od}}} + R_{\text{C}}W. \tag{8.2}$$

Here μ_{TLM} is the charge carrier mobility of the OSC in the conduction channel; V_{od} is the overdrive voltage, defined as $V_{\text{od}} = V_{\text{G}} - V_{\text{th}}$. In the limit of $L \to 0$, $R_{\text{tot}}W \to R_{\text{C}}W$. On practice this is achieved by characterizing devices with varying L, fixing V_{od}, plotting $R_{\text{tot}}W$ of each device as a function of L and fitting the dependence with a linear function. $R_{\text{C}}W$ is then given by the y-intercept of the linear fit, and the charge carrier mobility is extracted from the slope of the fit according to Equation 8.2. The extracted mobility is specifically denoted as μ_{TLM} to emphasize its distinction from the effective mobility μ_{eff}. The advantage of μ_{TLM} is that it does not depend on L and is therefore closer to representing the intrinsic mobility of the material. Furthermore, unlike μ_{eff}, which can be strongly affected by thin film inhomogeneity and/or imperfections of specific devices, μ_{TLM} has an "averaging" effect since it takes into account properties of a whole set of analyzed devices.

The TLM analysis is performed using the transfer characteristics in the linear regime of operation to derive the total resistance, i. e. $R_{\text{tot}}W(V_{\text{od}}) = V_{\text{D}}/I_{\text{D}}(V_{\text{od}})$. Since Equation 8.2 requires the total resistance at the same overdrive voltage, the $R_{\text{tot}}W(V_{\text{od}})$ curves are linearly interpolated at predefined values of V_{od}. The interpolation error is assumed to be negligible due to a small V_{od} step of 0.125 V to 0.15 V. The values of V_{od} are chosen such as not to extend outside of the range of the overdrive voltage overlap among the set of all measured transfer characteristics. The contact resistance $R_{\text{C}}W$ and the TLM charge carrier mobility μ_{TLM} are extracted for every V_{od} from the y-intercept and the slope, respectively, of the linear fit of the $R_{\text{tot}}W(L)$ curve, thus giving an overdrive voltage dependence for both quantities. The device

channel lengths used in the analysis are measured from optical micrographs of the individual devices to account for the discrepancy between the designed and actual channel length, which often leads to an overestimation of the contact resistance.[37] The $R_{\mathrm{C}}W(V_{\mathrm{od}})$ and $\mu_{\mathrm{TLM}}(V_{\mathrm{od}})$ dependencies, obtained in the variable-temperature analysis (see Section 4), are used in the Arrhenius analysis to extract the overdrive voltage dependence of the injection barrier Φ_{TE} (Equation 9.3) and the activation energy of the charge carrier transport E_{a} (Equation 9.5) at the highest common V_{od} by performing TLM analysis at each temperature as described above.

9 Arrhenius Analysis

The Arrhenius analysis allows to extract the injection barrier Φ_{TE} and the activation energy E_{a} from the temperature-dependent contact resistance and charge carrier mobility, respectively.

If the contact resistance is low, the charge carrier injection into the OSC can generally be described by the thermionic emission theory for Schottky diodes,[26, 38, 39] in which the contact resistance $R_{\mathrm{C}}W$ has an exponential dependence on the injection barrier Φ_{TE}:[40]

$$R_{\mathrm{C}}W = \left(\frac{k_B}{qA^*T}\right)\exp\left(\frac{\Phi_{\mathrm{TE}}}{k_{\mathrm{B}}T}\right), \tag{9.1}$$

here A^* – the Richardson constant. The advantage of using this model is that no assumptions regarding the height of the injection barrier are made in advance. Thus phenomena that directly affect the latter, such as the Schottky effect, are implicitly incorporated in the model.

Taking a logarithm of both sides of Equation 9.1, one gets a linear dependence on the inverse temperature:

$$\ln(R_{\mathrm{C}}W) \propto \Phi_{\mathrm{TE}}k_{\mathrm{B}}T^{-1}, \tag{9.2}$$

Fitting Equation 9.2 with a linear function allows extraction of the Schottky barrier from its slope:

$$\Phi_{\mathrm{TE}} = k_{\mathrm{B}}\frac{\partial \ln(R_{\mathrm{C}}W)}{\partial T^{-1}}. \tag{9.3}$$

The temperature dependence of the charge carrier mobility was analyzed within the framework of the hopping transport model, typically used for systems with a limited order, such as polycrystalline OSC films.[41] In hopping transport, the available states are energetically distributed and the charge transport occurs via transitioning ("hopping") from one localized state to another. The average energy required for a single transition is defined as the activation energy E_{a}. The charge carrier mobility then has a following dependence on the temperature:

$$\mu = \mu_0 \exp\left(-\frac{E_{\mathrm{a}}}{k_{\mathrm{B}}T}\right). \tag{9.4}$$

Here μ_0 is a temperature-independent prefactor. In a similar fashion to the Arrhenius analysis above, we logarithmize both sides of the equation to get a linear dependence on the inverse temperature T^{-1} and extract the activation energy from its slope:

$$E_{\mathrm{a}} = -k_{\mathrm{B}}\frac{\partial \ln(\mu)}{\partial T^{-1}} \tag{9.5}$$

It is worth noting that depending on the underlying physical transport mechanism, the proportionality in $\ln(\mu) \propto T^{-n}$ might not be linear. Indeed, the polaronic model yields a $\ln(\mu) \propto T^{-3/2}$ dependence,[42] while the Gaussian disorder model yields a $\ln(\mu) \propto T^{-2}$ dependence.[43] In our study for all datasets a $\ln(\mu) \propto T^{-1}$ was observed (see Figure 2(c) in the main text), consistent with the molecular geometry fluctuation model.[44]

10 Tabular Data

OSC	Surface	V_{th} [V]	ΔV_{th} [V]	S_{sub} [V dec^{-1}]	μ_{eff} a) [cm^2 V^{-1} s^{-1}]	μ_{TLM} b) [cm^2 V^{-1} s^{-1}]	$R_C W$ [kΩ cm]	WF [eV]	χ_{OSC} [eV]	Φ_{SM} [eV]
PEN	TDPA	$-6.67(53)^{-6.38}_{-7.03}$	$-0.69(18)^{-0.54}_{-0.87}$	$0.44(32)^{0.24}_{0.51}$	$0.39(19)^{0.20}_{0.53}$	0.99	6.78(36)	4.73(3)	5.07[45]	0.34(3)
C_8-DNTT	TDPA	$-6.27(54)^{-5.79}_{-6.55}$	$-0.51(12)^{-0.43}_{-0.56}$	$0.19(5)^{0.15}_{0.21}$	$3.14(1.36)^{1.88}_{4.48}$	5.77	0.44(3)	4.79(5)	5.40[46]	0.61(5)
PhC_2-BQQDI	TDPA	$-0.08(13)^{-0.01}_{-0.13}$	$0.04(2)^{+0.05}_{+0.02}$	$0.35(9)^{0.43}_{0.27}$	$0.45(6)^{0.49}_{0.39}$	0.79	4.57(97)	4.76(4)	4.11[18]	0.65(4)
PEN	Al_2O_3	$-1.34(42)^{-0.98}_{-1.63}$	$-0.73(22)^{-0.58}_{-0.83}$	$0.74(35)^{0.49}_{0.87}$	$0.03(1)^{0.02}_{0.04}$	0.11	207(42)	4.72(4)	5.07[45]	0.35(4)
C_8-DNTT c)	Al_2O_3	–	–	–	–	–	–	–	–	–
PhC_2-BQQDI	Al_2O_3	$2.82(40)^{+3.17}_{+2.51}$	$0.66(28)^{+0.79}_{+0.46}$	$1.24(16)^{1.35}_{1.16}$	$0.015(4)^{0.02}_{0.01}$	0.03	109(25)	4.78(5)	4.11[18]	0.67(5)

Table S1: Parameters extracted from the transfer characteristics in the linear regime of operation of OFETs measured at room temperature: threshold voltage V_{th}, threshold voltage hysteresis ΔV_{th}, subthreshold swing S_{sub}, effective mobility μ_{eff}, mobility extracted via the TLM analysis μ_{TLM} and width-normalized contact resistance $R_C W$; additionally, measured work function of the electrodes WF, transport level of the OSCs χ_{OSC} (from literature), and Schottky barrier estimated by the Schottky-Mott rule Φ_{SM}. Each set of measurements consisted of 13 to 28 devices with 5 to 10 different channel lengths. For all device parameters except ΔV_{th} the values extracted from forward and backward sweeps are averaged. The values are provided either in the form of $\left[\text{mean (standard deviation)}^{\text{3rd quartile}}_{\text{1st quartile}}\right]$, or in the form of [mean (standard deviation)]. Values of $R_C W$ and μ_{TLM} are taken at $V_{od} = 2.2$ V, the highest common overdrive voltage among all investigated systems at room temperature. For the work function WF a mean and a standard deviation of measurements at 3-4 different positions on the substrate is given. This error is then propagated to the Schottky-Barrier Φ_{SM}.

a) It should be mentioned that since μ_{eff} is generally dependent on the channel length L, it is expected that in presented datasets with L varying by up to two orders of magnitude, the variation of μ_{eff} is also significant.

b) Here the values for the uncertainty are not provided since the uncertainties reported by the utilized algorithm are two to seven orders of magnitude lower than the extracted value.

c) A reliable analysis of C_8-DNTT OFETs with a bare Al_2O_3 dielectric could not be performed due to a general poor performance of the devices and non-linearity of the transfer characteristics – see Section 5.

References

[1] A. Dürr, F. Schreiber, M. Kelsch, H. Carstanjen, H. Dosch, *Advanced Materials* **2002**, *14*, 961–963.

[2] Z. Bao, J. Locklin, *Organic Field-Effect Transistors*, CRC Press, Boca Raton, **2007**, 616 pp.

[3] I. Kymissis, *Organic Field Effect Transistors: Theory, Fabrication and Characterization*, Springer, New York, NY, **2009**, 148 pp.

[4] Z. A. Lamport, H. F. Haneef, S. Anand, M. Waldrip, O. D. Jurchescu, *Journal of Applied Physics* **2018**, *124*, 071101.

[5] U. Zschieschang, F. Ante, M. Schlörholz, M. Schmidt, K. Kern, H. Klauk, *Advanced Materials* **2010**, *22*, 4489–4493.

[6] R. Jagdheesh, *Langmuir* **2014**, *30*, 12067–12073.

[7] M. Geiger, R. Acharya, E. Reutter, T. Ferschke, U. Zschieschang, J. Weis, J. Pflaum, H. Klauk, R. T. Weitz, *Advanced Materials Interfaces* **2020**, *7*, 1902145.

[8] D. Nečas, P. Klapetek, *Open Physics* **2012**, *10*, 181–188.

[9] D. Nečas in *Quantitative Data Processing in Scanning Probe Microscopy (Second Edition)*, (Ed.: P. Klapetek), Micro and Nano Technologies, Elsevier, **2018**, pp. 65–96.

[10] M. Pfeiffer, K. Leo, N. Karl, *Journal of Applied Physics* **1996**, *80*, 6880–6883.

[11] G. N. Derry, M. E. Kern, E. H. Worth, *Journal of Vacuum Science & Technology A* **2015**, *33*, 060801.

[12] Y. Radiev, F. Widdascheck, M. Göbel, A. A. Hauke, G. Witte, *Organic Electronics* **2021**, *89*, 106030.

[13] No digital integration with 10 points per sweep. The actual measurement is performed with averaging over 16 samples taken during one line frequency period for each point, with 80 points per sweep.

[14] *IEEE Standard for Test Methods for the Characterization of Organic Transistors and Materials*, **2008**.

[15] U. Zschieschang, R. T. Weitz, K. Kern, H. Klauk, *Applied Physics A* **2009**, *95*, 139–145.

[16] U. Zschieschang, F. Ante, D. Kälblein, T. Yamamoto, K. Takimiya, H. Kuwabara, M. Ikeda, T. Sekitani, T. Someya, J. B. Nimoth, H. Klauk, *Organic Electronics* **2011**, *12*, 1370–1375.

[17] M. J. Kang, I. Doi, H. Mori, E. Miyazaki, K. Takimiya, M. Ikeda, H. Kuwabara, *Advanced Materials* **2011**, *23*, 1222–1225.

[18] Toshihiro Okamoto, Shohei Kumagai, Eiji Fukuzaki, Hiroyuki Ishii, Go Watanabe, Naoyuki Niitsu, Tatsuro Annaka, Masakazu Yamagishi, Yukio Tani, Hiroki Sugiura, Tetsuya Watanabe, Shun Watanabe, Jun Takeya, *Science Advances* **2020**, *6*, eaaz0632.

[19] R. Acharya, D. Günder, T. Breuer, G. Schmitz, H. Klauk, G. Witte, *Journal of Materials Chemistry C* **2021**, *9*, 270–280.

[20] T. Minari, T. Nemoto, S. Isoda, *Journal of Applied Physics* **2006**, *99*, 034506.

[21] M. J. Kang, E. Miyazaki, I. Osaka, K. Takimiya, A. Nakao, *ACS Applied Materials & Interfaces* **2013**, *5*, 2331–2336.

[22] J. Milvich, T. Zaki, M. Aghamohammadi, R. Rödel, U. Kraft, H. Klauk, J. N. Burghartz, *Organic Electronics* **2015**, *20*, 63–68.

[23] J. Smith, R. Hamilton, Y. Qi, A. Kahn, D. D. C. Bradley, M. Heeney, I. McCulloch, T. D. Anthopoulos, *Advanced Functional Materials* **2010**, *20*, 2330–2337.

[24] V. Podzorov, E. Menard, A. Borissov, V. Kiryukhin, J. A. Rogers, M. E. Gershenson, *Physical Review Letters* **2004**, *93*, 086602.

[25] H. F. Haneef, A. M. Zeidell, O. D. Jurchescu, *Journal of Materials Chemistry C* **2020**, *8*, 759–787.

[26] S. Sze, K. N. Kwok, *Physics of Semiconductor Devices*, 3rd ed., John Wiley & Sons, Ltd, Hoboken, NJ, **2006**.

[27] M. Grünewald, P. Thomas, D. Würtz, *physica status solidi (b)* **1980**, *100*, K139–K143.

[28] G. Fortunato, D. B. Meakin, P. Migliorato, P. G. Le Combers, *Philosophical Magazine B* **1988**, *57*, 573–586.

[29] G. Horowitz, R. Hajlaoui, P. Delannoy, *Journal de Physique III* **1995**, *5*, 355–371.

[30] P. Servati, D. Striakhilev, A. Nathan, *IEEE Transactions on Electron Devices* **2003**, *50*, 2227–2235.

[31] D. V. Lang, X. Chi, T. Siegrist, A. M. Sergent, A. P. Ramirez, *Physical Review Letters* **2004**, *93*, 086802.

[32] W. L. Kalb, F. Meier, K. Mattenberger, B. Batlogg, *Physical Review B* **2007**, *76*, 184112.

[33] D. Oberhoff, K. P. Pernstich, D. J. Gundlach, B. Batlogg, *IEEE Transactions on Electron Devices* **2007**, *54*, 17–25.

[34] W. L. Kalb, B. Batlogg, *Physical Review B* **2010**, *81*, 035327.

[35] M. Geiger, L. Schwarz, U. Zschieschang, D. Manske, J. Pflaum, J. Weis, H. Klauk, R. T. Weitz, *Physical Review Applied* **2018**, *10*, 044023.

[36] M. Waldrip, O. D. Jurchescu, D. J. Gundlach, E. G. Bittle, *Advanced Functional Materials* **2020**, *30*, 1904576.

[37] T. Wollandt, S. Steffens, Y. Radiev, F. Letzkus, J. N. Burghartz, G. Witte, H. Klauk, *ACS Nano* **2025**, *19*, 9915–9924.

[38] C. Liu, Y. Xu, Y.-Y. Noh, *Materials Today* **2015**, *18*, 79–96.

[39] A. Allain, J. Kang, K. Banerjee, A. Kis, *Nature Materials* **2015**, *14*, 1195–1205.

[40] J. W. Borchert, R. T. Weitz, S. Ludwigs, H. Klauk, *Advanced Materials* **2022**, *34*, 2104075.

[41] R. Noriega, A. Salleo in *Organic Electronics II*, John Wiley & Sons, Ltd, **2012**, pp. 67–104.

[42] V. Coropceanu, J. Cornil, D. A. da Silva Filho, Y. Olivier, R. Silbey, J.-L. Brédas, *Chemical Reviews* **2007**, *107*, 926–952.

[43] H. Bässler, *physica status solidi (b)* **1993**, *175*, 15–56.

[44] Z. G. Yu, D. L. Smith, A. Saxena, R. L. Martin, A. R. Bishop, *Physical Review Letters* **2000**, *84*, 721–724.

[45] P. G. Schroeder, C. B. France, J. B. Park, B. A. Parkinson, *Journal of Applied Physics* **2002**, *91*, 3010–3014.

[46] K. Takimiya, S. Shinamura, I. Osaka, E. Miyazaki, *Advanced Materials* **2011**, *23*, 4347–4370.
